# Supplementary material for: CD36 facilitates fatty acid uptake by dynamic palmitoylation-regulated endocytosis
Source: Nat Commun. 2020 Sep 21;11:4765. doi: 10.1038/s41467-020-18565-8 (PMC7505845; doi:10.1038/s41467-020-18565-8)
Supplement: Supplementary file 1 — Supplementary Information [file 41467_2020_18565_MOESM1_ESM.pdf]

Supplementary Information

CD36 Facilitates Fatty Acid Uptake by Dynamic Palmitoylation-Regulated Endocytosis

Hao et al.

Supplementary Figure 1

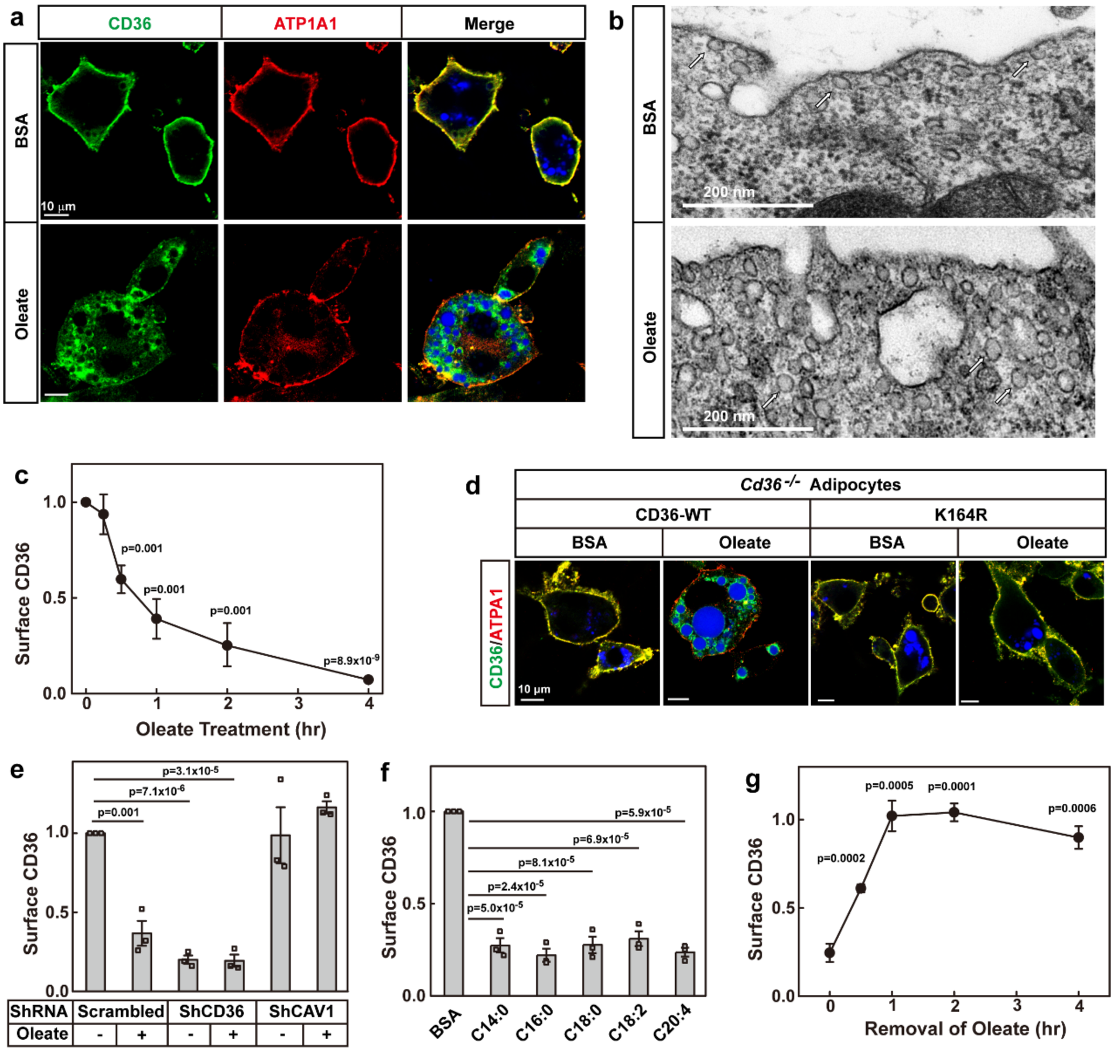

Supplementary Figure 1. Long-chain fatty acids trigger internalization of CD36

a, 3T3-L1 adipocytes were treated as in Fig. 1a with BSA or BSA-conjugated oleate (100 μM) for 4 hr. Cells were harvested and subjected to immunostaining using anti-CD36 and anti-ATP1A1 antibodies. **b,** 3T3-L1 adipocytes were treated with BSA-conjugated oleate (100 μM) for 1 hr and subjected to electron microscopy analysis. Arrows indicated representative caveolaes in each picture. **c,** Quantification of surface content of CD36 in Fig. 1b. Each value represents mean ± SEM from 3 independent experiments. The value in BSA-treated cells was normalized to 1.0. Two-sided

Student's t test was performed. **d,** SVFs were isolated from iWAT of *Cd36*$^{-/-}$ mice. On Day 6 of differentiation, cells were electroporated with WT or K164R mutant of CD36/pcDNA3.3. On day 7, cells were treated as in Fig. 1a with BSA-conjugated oleate (100 μM) for 1 hr, and subjected to immunostaining as in (a). LipidTOX (blue) was used to indicate lipid droplet. **a, d,** The scale bars were as indicated. **e-g,** Quantification of surface CD36 content in Fig. 1d (e), Fig.1 f (f) and Fig. 1h (g), respectively. Each value represents mean ± SEM from 3 independent experiments. The value in BSA-treated cells was normalized to 1.0. Two-sided Student's t test was performed. The experiment was repeated at least twice. Source data are provided as a Source Data file.

Supplementary Figure 2

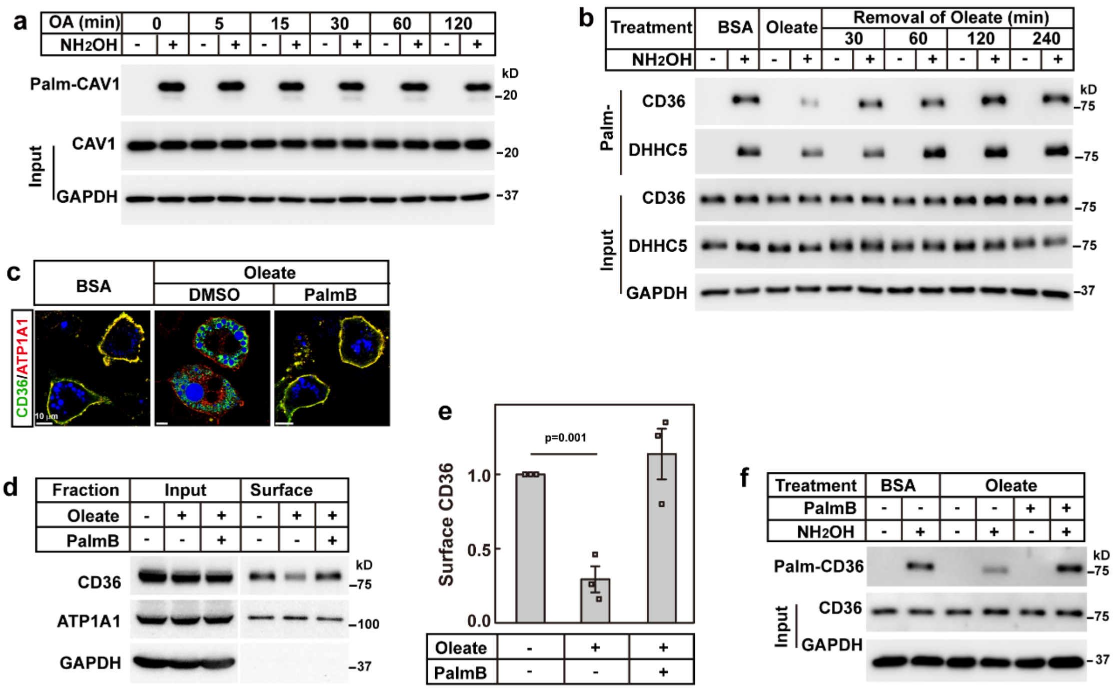

Supplementary Figure 2. Depalmitoylation of CD36 is required for its internalization

a, 3T3-L1 adipocytes were treated and subjected to Acyl-RAC as in Fig. 3a, followed by Western blot with indicated antibodies. **b,** 3T3-L1 adipocytes were treated with oleate for 4 hr, and then switched to serum free medium for indicated time. Cells were harvested for Acyl-RAC analysis. **c-f,** 3T3-L1 adipocytes were pretreated with serum free medium for 4 hr, and DMSO or PalmB (15 μM) for 1 hr, followed by BSA or oleate (100 μM) treatment for 1 hr. Cells were harvested for immunostaining (c), surface biotinylation (d, e) and Acyl-RAC (e) assays. **e,** Each value represents mean ± SEM from 3 independent experiments. The value in BSA-treated cells was normalized to 1.0. Two-sided Student's t test was performed between BSA- and oleate-treated cells. These experiments were repeated at least twice. Source data are provided as a Source Data file.

Supplementary Figure 3

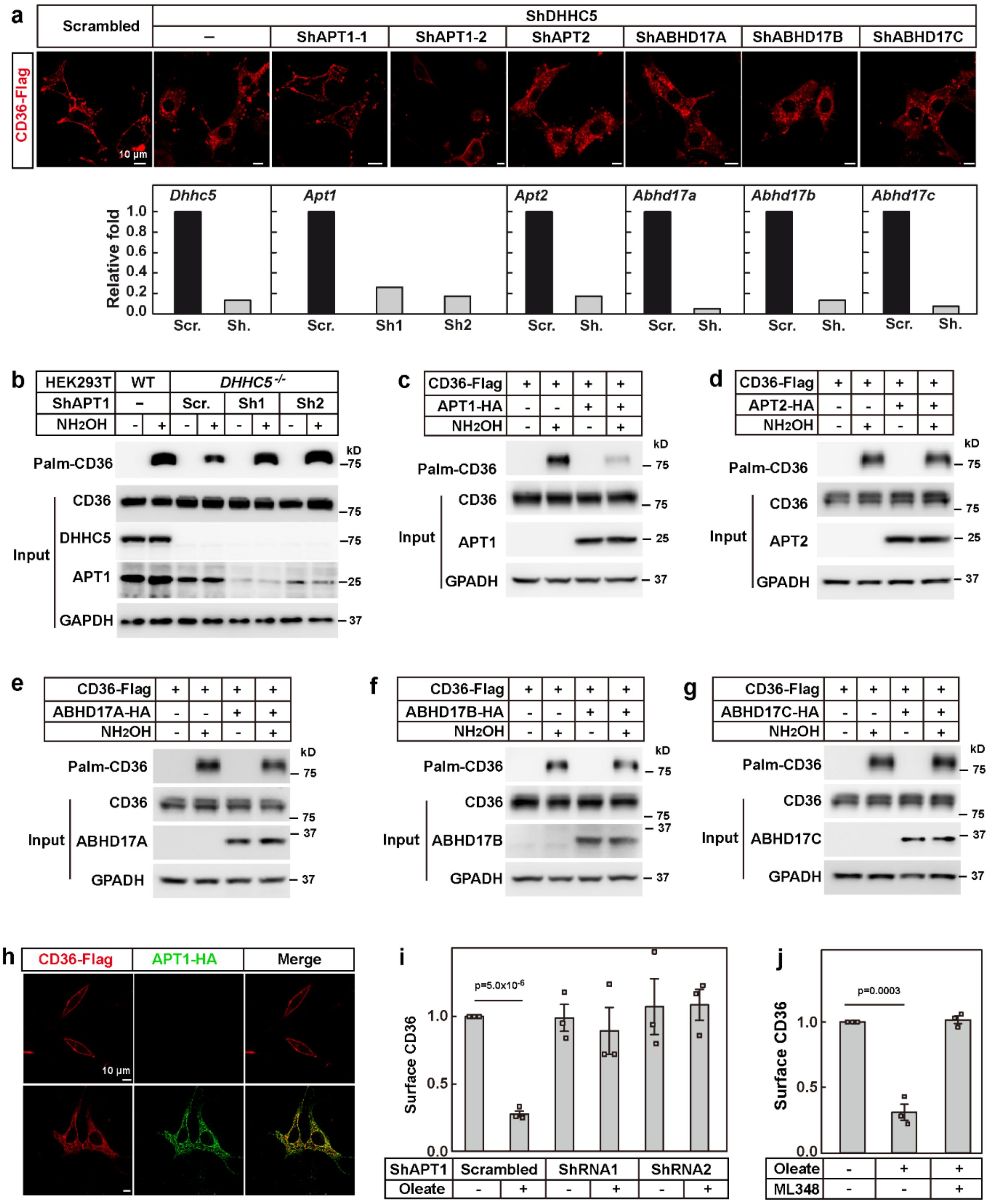

Supplementary Figure 3. Identification of APT1 as the depalmitoylase of CD36

a, On day 0, 3T3-L1 preadipocytes transduced with indicated shRNAs were set up at 4×10^4 cells per well in a 6-well plate. On day 2, cells were infected with lentivirus-expressing CD36-Flag. On day 3, cells were harvested for immunostaining with anti-Flag antibody. The knockdown efficiency of each shRNA was shown in the lower panel.

b, On day 0, WT or *DHHC5*$^{-/-}$ HEK293T cells were infected with scrambled shRNA or each of the two shRNAs of APT1. On day 1, cells were selected with 1 μg/ml puromycin. On day 3, cells were transfected with 1 μg CD36-Flag/pCDH-puro. On day 4, cells were harvested for Acyl-RAC assay and blotted with indicated antibodies. **c-g,** On day 0, HEK-293T cells were set up at 7.5 × 10^5 cells per 6-cm dish. On day 2, cells were transfected with 0.5 μg CD36-Flag/pCDH-puro and/or 0.5 μg of each of the depalmitoylases on a pCDH-puro vector. On day 3, cells were harvested for Acyl-RAC assay and blotted with indicated antibodies. **h,** On day 0, 3T3-L1 preadipocytes were set up at 4 × 10^4 cells per well in a 6-well plate. On day 2, cells were infected with lentivirus-expressing CD36-Flag with or without APT1-HA. On day 3, cells were harvested for immunostaining with anti-Flag and anti-HA antibodies. The scale bars were as indicated. **i,j,** Quantification of surface CD36 content in Fig. 3c (i) and Fig. 3f (j). Each value represents mean ± SEM of a triplicate. The value in BSA-treated cells was normalized to 1.0. Two-sided Student's t test was performed. These experiments were repeated at least twice. Source data are provided as a Source Data file.

Supplementary Figure 4

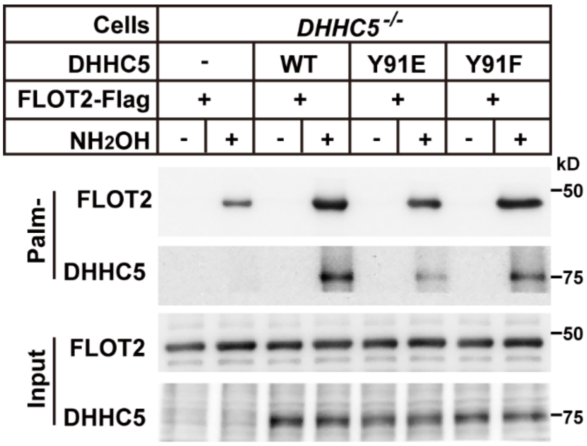

Supplementary Figure 4. Y91E mutant of DHHC5 showed decreased activity in palmitoylating Flotillin-2.

DHHC5$^{-/-}$ HEK-293T cells were set up, transfected and subjected to Acyl-RAC analysis as in Fig. 4f, except that Flotillin-2 (FLOT2) was used as a substrate. This experiment was repeated twice. Source data are provided as a Source Data file.

Supplementary Figure 5

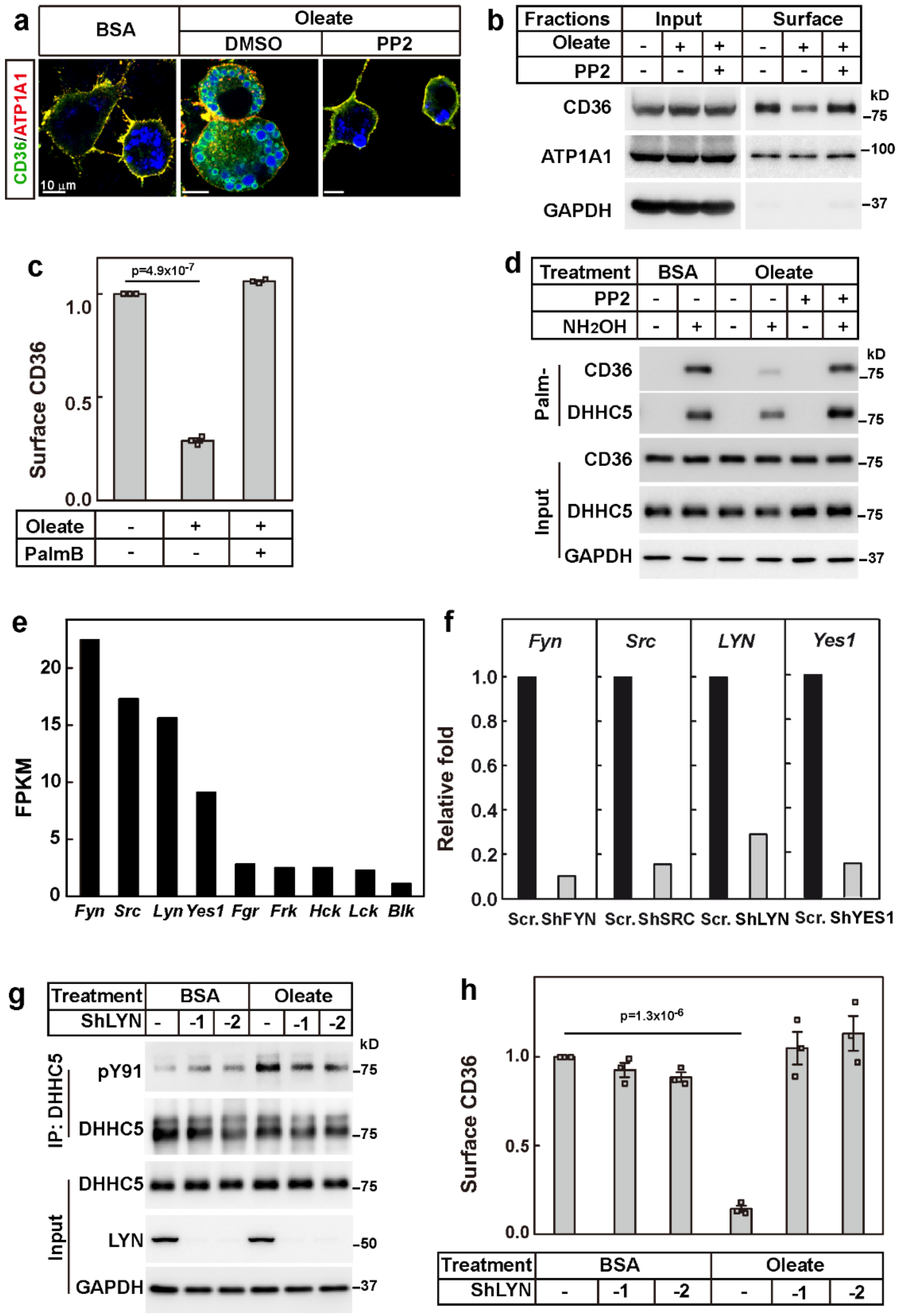

Supplementary Figure 5. LYN phosphorylates DHHC5

a-d, 3T3-L1 adipocytes were pretreated with serum free medium for 4 hr and PP2 (15 µM) for 1 hr, followed by treatment with BSA or oleate (100 µM) for 1 hr. Cells were

harvested for immunostaining (a), surface biotinylation (b, c) and Acyl-RAC (d) assays. **c,** Each value represents mean ± SEM of 3 individual experiments. The value in BSA-treated cells was normalized to 1.0. Two-sided Student's t test was performed. **e,** Expression pattern of SRC family kinases in white adipose tissue. These data were from a RNA-Seq in the white adipose tissue from 8-week-old male C57BL6 mice. **f,** Knockdown efficiency of the shRNAs against indicated SFKs. **g,** Control and LYN knockdown 3T3-L1 adipocytes were pretreated and treated with oleate for 5 min, followed by immunoprecipitation of DHHC5 to detect phosphorylation of DHHC5 (pY91). **h,** Quantification of surface CD36 content in Fig. 5e. Each value represents mean ± SEM of 3 individual experiments. The value in BSA-treated cells was normalized to 1.0. Two-sided Student's t test was performed. These experiments were repeated at least twice. Source data are provided as a Source Data file.

Supplementary Figure 6

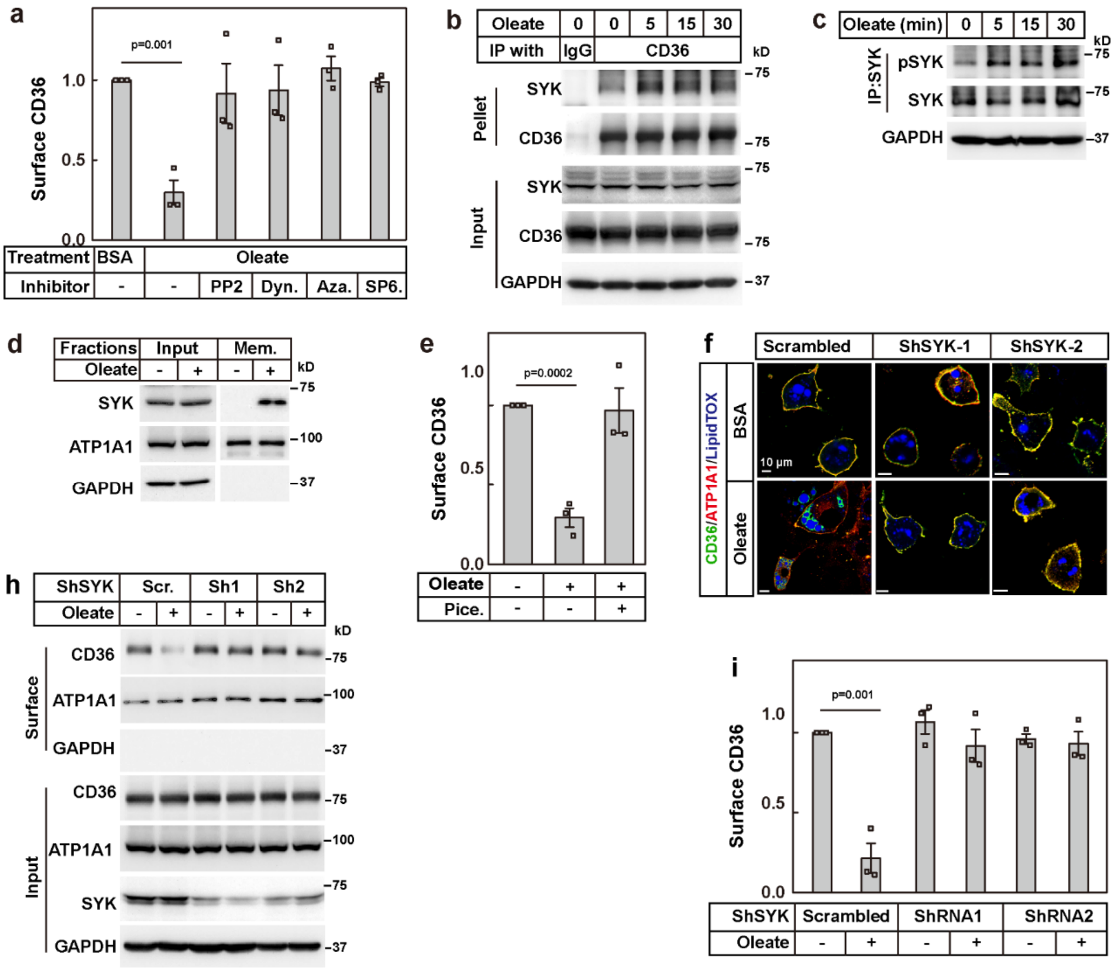

Supplementary Figure 6. SYK is required for oleate-induced internalization of CD36

a, Quantification of surface CD36 content in Fig. 6b. Each value represents mean ± SEM from 3 independent experiments. The value in BSA-treated cells was normalized to 1.0. Two-sided Student's t test was performed. **b,** 3T3-L1 adipocytes were pretreated and treated with BSA-conjugated oleate (100 μM) for indicated. Cells were harvested for immunoprecipitation with anti-CD36 and blotted with indicated antibodies. **c,** 3T3-L1 adipocytes were pretreated and treated as in (b). Cells were harvested, subjected to immunoprecipitation with anti-SYK antibody, and blotted with anti-SYK

and anti-pSYK (Y525/526). **d,** 3T3-L1 adipocytes were pretreated and treated with BSA-conjugated oleate (100 μM) for 5 min. Cells were harvested and membrane fractions were collected for immunoblotting using the indicated antibodies. **e,** Quantification of surface CD36 content in Fig. 6h. Each value represents mean ± SEM from 3 independent experiments. The value in BSA-treated cells was normalized to 1.0. Two-sided Student's t test was performed. **f-i,** Control and SYK knockdown cells were pretreated and treated with oleate as in Fig. 3b. Cells were harvested for immunostaining (f) and surface biotinylation (h,i) assays. **i,** Quantification of surface CD36 content in Fig. 6b. Each value represents mean ± SEM from 3 independent experiments. The value in BSA-treated cells was normalized to 1.0. Two-sided Student's t test was performed between BSA- and oleate-treated cells. These experiments were repeated at least twice. Source data are provided as a Source Data file.

Supplementary Figure 7

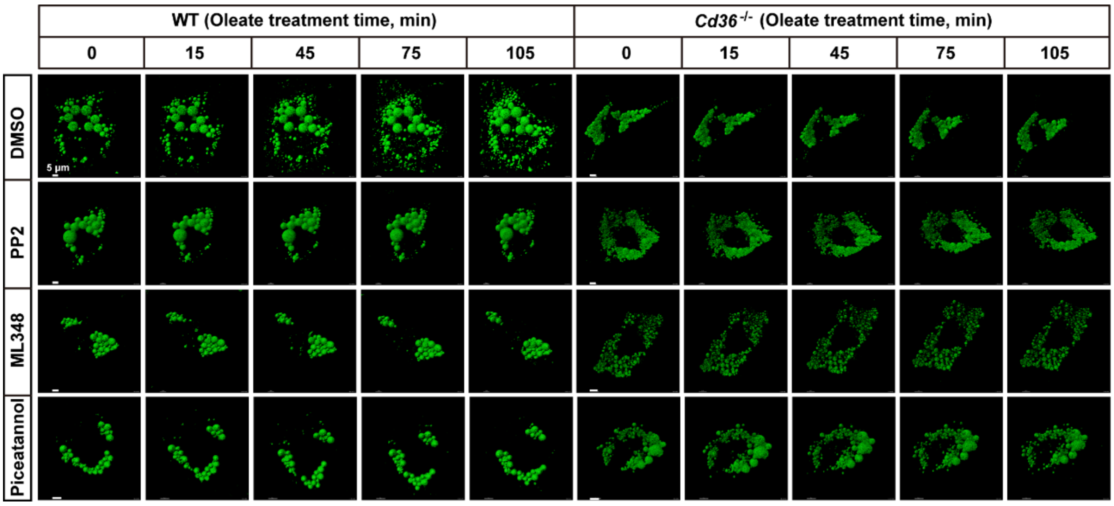

Supplementary Figure 7. Block endocytosis inhibits CD36-dependent lipid droplet growth

Representative images of Fig. 7a. Images were reconstructed with Imaris 9.2.0. Scale bar, 5 μm. This experiment was repeated twice.

Supplementary Figure 8

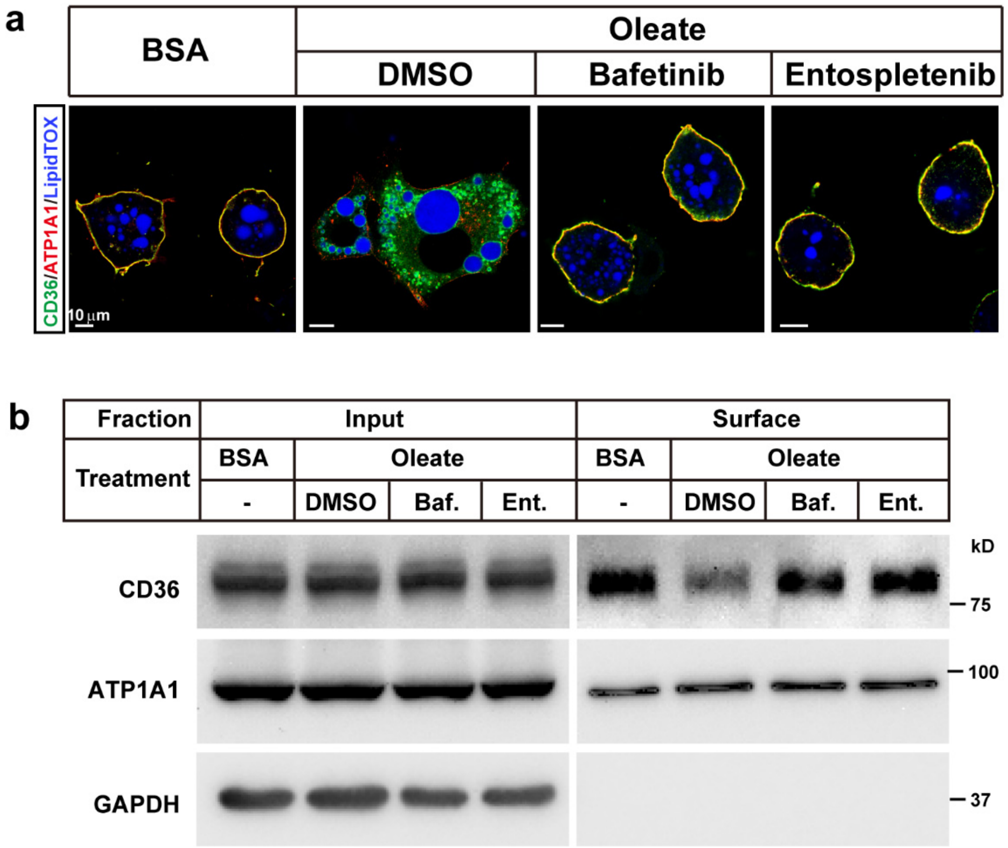

Supplementary Figure 8. Bafetinib and entospletenib blocks oleate-induced endocytosis of CD36

a, b, 3T3-L1 adipocytes were set up, pretreated and treated as in Fig. 6A, except that cells were pretreated with bafetinib (Baf., 10 μM) or entospletenib (Ent., 5 μM) for 1 hr. Cells were harvested for immunostaining (a) and surface biotinylation (b). This experiment was repeated twice. Source data are provided as a Source Data file.

Supplementary Figure 9

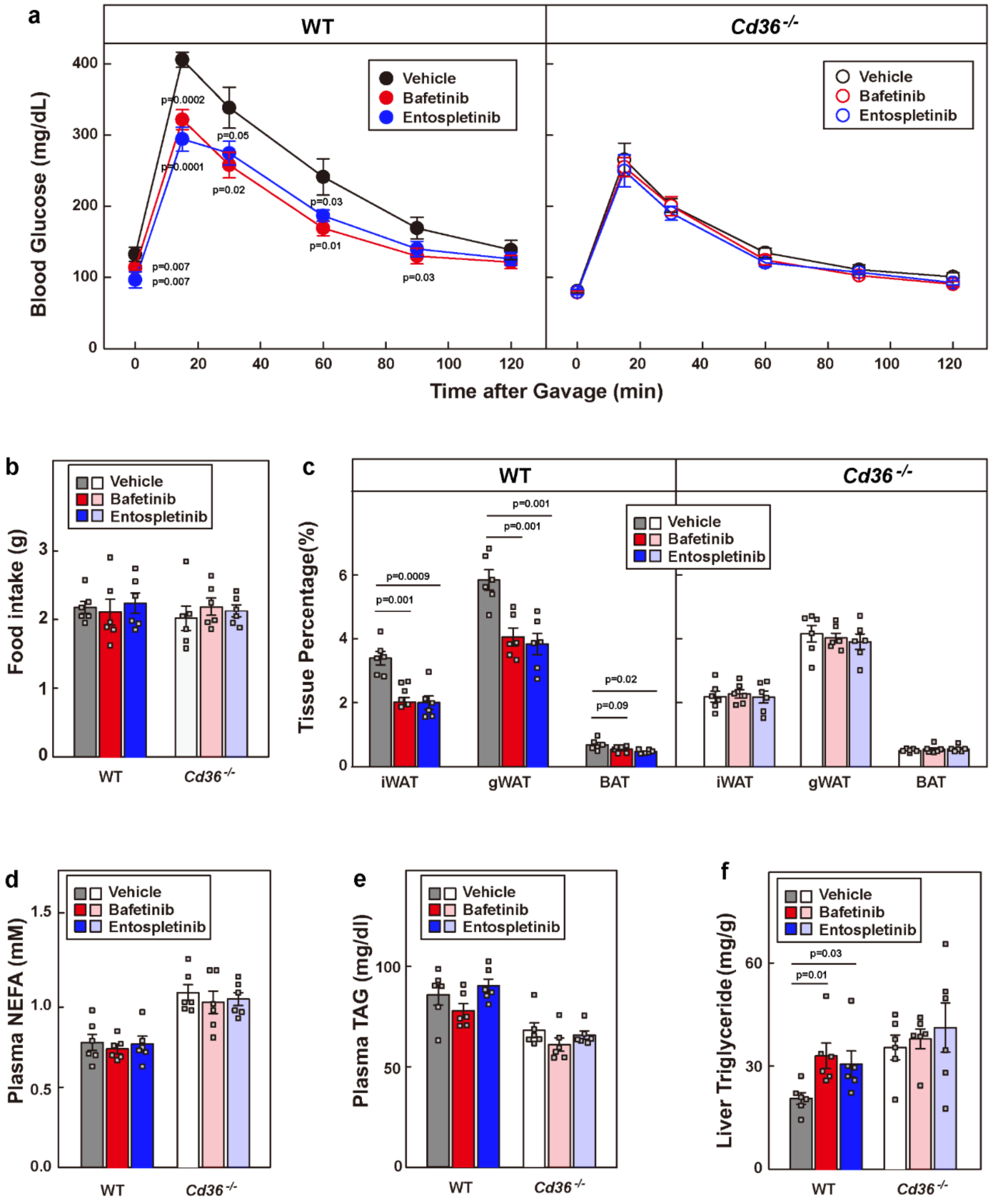

Supplementary Figure 9. Blocking the endocytosis protects mice from HFD-induced obesity

a-f, The mice used in this experiment were the same with Fig. 7b. **a,** On week 8, mice were fasted from 5 pm to 9 am for 16 hr, and orally gavaged with glucose at 1 mg/kg body weight. Blood glucose levels were measured at indicated time from the tail vein.

Two-sided Student's t test was performed between vehicle- and bafetinib or entospletinib-treated cells. **b,** Mice were housed individually for 2 days before food intake measurement for another 4 days. For each mouse, the food intake was a mean from the 4-day experiment. **c,** Tissues were collected and the percentage of each tissue to body weight was plotted. **d-f,** At the end of the experiment, plasma non-esterified fatty acids (d), plasma triglyceride (e) and liver triglyceride (f) were measured. **a-f,** Each value represents mean ± SEM from 6 mice. Two-sided Student's t test was performed. This experiment was repeated twice. Source data are provided as a Source Data file.

Supplementary Table 1. Primer information

Primers	Source of Primer sequences
**A. Primers to generate different constructs**	
mLYN-F	CAGA GCTAGCATGGGATGTATTAAATCAAAAAG
mLYN-R	TCTGCTCGAGCGGTTGCTGCTGATACTGCCC
hDHHC5-F	CAGAGAATTCATGCCCGCAGAGTCTGGAAAG
hDHHC5-R	GCTGGGATCC CACCGAAATC TCATAGGTGG
hDHHC5-Y91E-F	GCTCCCCTTGAGAAAACAGTGGAGATAAAGGG
hDHHC5-Y91E-R	CACTGTTTTCTCAAGGGGAGCTCGGAAATCATC
hDHHC5-Y91F-F	GCTCCCCTTTTCAAAACAGTGGAGATAAAGGG
hDHHC5-Y91F-R	CACTGTTTTGAAAAGGGGAGCTCGGAAATCATC
mAPT1-shRNA1	CCCTAATGTTTGGTTCTCTTA
mAPT1-shRNA2	GCAGGAAATGATGGATGTCAA
mABHD17A-shRNA1	GAAGAACCTCTATGCTGACAT
mABHD17B-shRNA1	GCATTCCCAAACATTGACAAA
mABHD17C-shRNA1	GCACAGTACCTAGAACGACTA
m APT2-shRNA	GAGAACATCAAGGCTTTGATT
hAPT1-shRNA1	CTATGCCTTCATGGTTTGATA
hAPT1-shRNA2	CTATGCCTTCATGGTTTGATA
mCAV1-shRNA	GCTTCCTGATTGAGATTCAGT
mCD36-shRNA	CGGATCTGAAATCGACCTTAA
mLYN-shRNA1	CGCGAGAGTCATCGAAGATAA
mLYN-shRNA2	CGCGAGAGTCATCGAAGATAA
mSRC-shRNA	CCTAAATGTGAAACACTACAA
mYES1-shRNA	GCTGCTCTGTATGGTCGATTT
mFYN-shRNA	CCTGTATGGAAGGTTCACAAT
mSYK-shRNA1	GCAGCAGAACAGGCACATTAA
mSYK-shRNA2	GGAACTGAGGCTTCGCAATTA
**B. Quantitative real-time PCR Primers**	
m*Lypla1*	TTTTCCTTCACGGATTGGGAG;<br>GGGGACTTTTGATACCTGCAA
m*Lypla2*	ATGTGTGGTAACACCATGTCTG;<br>ACTCAGCCCCATCAGGTCAA
m*Abhd17a*	CTCCCGATCCCACCTACTCTC;<br>GGCCGTACTGGAAGTCAGC
m*Abhd17b*	TGGCCTCGCATTGTTTGAG;<br>TTCCTGTGACACGAACTGTTTTA
m*Abhd17c*	TCTTACGACTACTCGGGCTATG;<br>CAACACGCAAACCAGACATCA
m*Lyn*	GTGACATTGTGGTGGCCTTAT;<br>ACCATTCCCCATGCTCTTCTA
m*Src*	GAACCCGAGAGGGACCTTC;<br>GAGGCAGTAGGCACCTTTTGT

m*Yes1*	AGTCCAGCCATAAAATACACACC;<br>TGATGCTCCCTTTGTGGAAGA
m*Fyn*	ACCTCCATCCCGAACTACAAC;<br>CGCCACAAACAGTGTCACTC
